# Assessing the Impact of Neuromuscular Taping on Thrombocyte Indices in Diabetic Neuropathy Patients With Peripheral Artery Disease: A Cross‐Sectional Study

**DOI:** 10.1002/hsr2.70919

**Published:** 2025-07-20

**Authors:** Nurul Aktifah, Firman Faradisi, Muhammad Ghilang Maulud Setyawan, Nuniek Nizmah Fajriyah, Eko Mugiyanto, Umi Budi Rahayu

**Affiliations:** ^1^ Undergraduate Program in Nursing University of Muhammadiyah Pekajangan Pekalongan Pekalongan Indonesia Indonesia; ^2^ Vocational Program in Nursing University of Muhammadiyah Pekajangan Pekalongan Pekalongan Indonesia Indonesia; ^3^ Undergraduate Program in Physiotherapy University of Muhammadiyah Pekajangan Pekalongan Pekalongan Indonesia Indonesia; ^4^ Professional Education Program in Pharmacy University of Muhammadiyah Pekajangan Pekalongan Pekalongan Indonesia; ^5^ Faculty of Health Sciences Universitas Muhammadiyah Surakarta Surakarta Indonesia

**Keywords:** diabetic neuropathy, genomic analysis, NMT intervention, platelet indices, TCNSS

## Abstract

**Background and Aims:**

Peripheral artery disease (PAD) is a common complication among diabetic neuropathy patients, often associated with abnormalities in thrombocyte indices. This study aimed to assess the impact of neuromuscular taping (NMT) on thrombocyte indices in diabetic neuropathy patients with PAD.

**Methods:**

A total of 23 patients diagnosed with DN using the Toronto Clinical Neuropathy Scoring System (TCNSS) and Diabetic Neuropathy Examination (DNE) were enrolled in the study. Participants underwent NMT decompression intervention sessions over a period of 24 days. Further, the genomic analysis utilized public databases derived from diabetes studies that investigated the development of diabetic neuropathy.

**Results:**

The result revealed, pre‐ and post‐intervention measurements demonstrated significant improvements in thrombocyte indices, particularly in platelet distribution width, platelet count, and mean platelet volume, among patients undergoing NMT (*p* < 0.05). Additionally, there was a notable decrease in TCNSS and DNE scores post‐intervention, indicating an improvement in DN symptoms. Moreover, genomic analysis identified 9 genes, including SLC30A1, TRBJ2‐7, OLFM1, TCF7L2, MCF2L, CEP295NL, CEACAM22P, TSHZ2, and PDZD4, involved in DN development.

**Conclusion:**

These findings suggest that NMT holds promise as a therapeutic intervention for improving thrombocyte indices and managing DN symptoms in patients with PAD. Further research is warranted to elucidate the underlying mechanisms and long‐term effects of NMT in this population.

**Abbreviations**
ABI: ankle‐brachial index
AGEs: Advanced Glycation End‐Products
BMI: body mass index
CEACAM22P: CEA cell adhesion molecule 22, pseudogene
CEP295NL: CEP295 N‐Terminal Like
DEGs: Differential Expression Genes
DM: Diabetes Mellitus
DN: Diabetic Neuropathy
DNE: Diabetic Neuropathy Examination
DNS: Diabetic Neuropathy Syndrome
EDTA: Ethylene Diamine Tetra Acetic Acid
GSK3: Glycogen synthase kinase‐3
MCF2L: MCF.2 Cell Line Derived Transforming Sequence‐like
MPV: Mean Platelet Volume
MPV: Mean Platelet Volume
NF‐κB: Nuclear Factor Kappa B
NMT: Neuromuscular Taping
OLFM1: Olfactomedin 1
PAD: peripheral artery disease
PARP: Poly (ADP‐ribose) polymerase
PCT: Plateletcrit
PDW: Platelet Distribution Width
PDZD4: PDZ domain containing 4
PKCs: Protein kinase C
SLC30A1: solute carrier family 30 member 1
T2DM: Type 2 Diabetes Mellitus
TCF7L2: transcription factor 7 like 2
TCNSS: Toronto Clinical Neuropathy Scoring System
TNF‐α: Tumor Necrosis Factor Alpha
TRBJ2‐7: Gene ‐ T cell receptor beta joining 2‐7
TSHZ2: teashirt zinc finger homeobox 2

## 1 Introduction

The chronic elevation of blood sugar levels in diabetes expedites the progression of atherosclerosis, a condition characterized by the buildup of fatty deposits within the walls of arteries [1]. Peripheral artery disease frequently occurs as a complication in individuals suffering from DN, significantly complicating disease management and exacerbating associated health challenges [2]. The coexistence of PAD with DN poses substantial obstacles in effectively addressing both conditions, leading to heightened health risks and complications for affected individuals. Patients with both PAD and DN face an increased burden of disease management, as they must navigate the intricate interplay between chronic hyperglycemia, atherosclerosis, and peripheral nerve damage [3, 4]. Not only the progression of atherosclerosis exacerbates the risk of developing PAD but also contributes to the impairment of peripheral nerve function, leading to diabetic neuropathy. Consequently, individuals with PAD and DN experience a compounding of symptoms, including intermittent claudication, neuropathic pain, and heightened susceptibility to foot ulcers and infections [5, 6]. Moreover, the presence of PAD further complicates the management of diabetes and its associated complications. Peripheral artery disease diminishes blood flow to the lower extremities, impairing wound healing and increasing the risk of tissue necrosis and amputation in diabetic patients [7, 8]. Additionally, the compromised arterial circulation exacerbates diabetic neuropathy symptoms, amplifying neuropathic pain and sensory deficits in the lower limbs [9]. This dual burden places a considerable strain on patients' physical and psychological well‐being, often necessitating complex treatment regimens and intensive healthcare interventions.

Among the array of management strategies available, NMT emerges as a promising therapeutic intervention deserving attention. NMT involves the application of specialized elastic tapes to specific areas of the body to facilitate neuromuscular function, enhance blood circulation, and alleviate pain. By strategically applying NMT techniques, healthcare providers can target underlying neuromuscular dysfunctions and vascular impairments commonly observed in patients with PAD and DN. This innovative approach not only complements traditional medical treatments but also offers unique benefits in improving muscle function, reducing pain perception, and enhancing overall mobility and physical well‐being. In conjunction with NMT, healthcare providers must also integrate other evidence‐based interventions into the comprehensive management plan for patients with PAD and DN. This may include pharmacological, promote cardiovascular health, alleviate neuropathic pain, and lifestyle modifications. Furthermore, various molecular pathways, such as the polyol pathway, hexosamine pathway, PKCs signaling, oxidative stress, AGEs pathway, PARP pathway, MAPK pathway, NF‐κB signaling, hedgehog pathways, TNF‐α signaling, cyclooxygenase pathway, interleukins, lipoxygenase pathway, nerve growth factor, Wnt pathway, autophagy, and GSK3 signaling, are implicated in the development and advancement of diabetic neuropathy [10, 11]. Despite the extensive literature on the molecular mechanisms of diabetic neuropathy, research on hematological molecular mechanisms remains limited.

Thrombocyte indices play pivotal roles in vascular health and can serve as indicators of cardiovascular risk in patients with DN and PAD [12, 13]. NMT has emerged as a promising therapeutic intervention in various medical fields, demonstrating efficacy in improving muscle function, reducing pain, and enhancing blood circulation [14, 15]. However, the impact of NMT on thrombocyte indices in diabetic neuropathy patients with peripheral artery disease remains understudied. Understanding the potential effects of NMT on thrombocyte indices could provide valuable insights into its role in managing vascular complications and improving overall patient outcomes in this population. Therefore, this study aims to assess the impact of neuromuscular taping on thrombocyte indices in diabetic neuropathy patients with peripheral artery disease.

## 2 Methods

**Figure 1:**
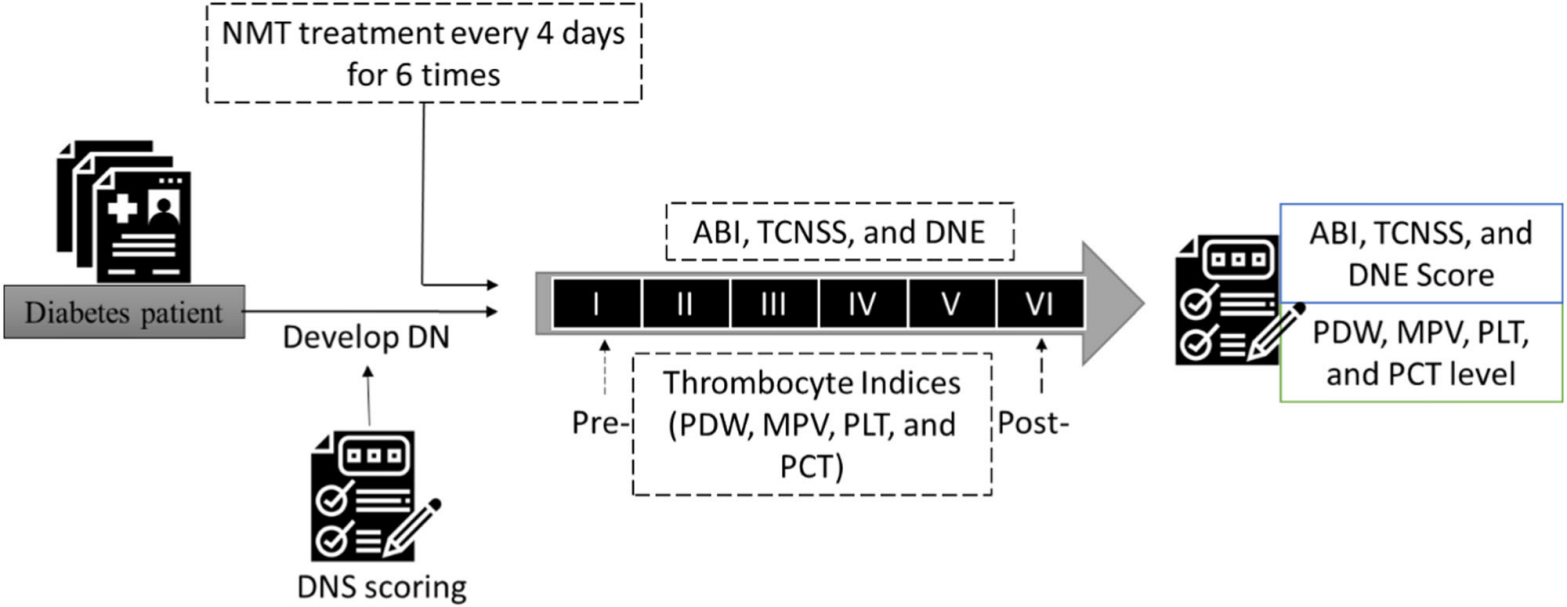
Schematic diagram of the study.

The research utilized a quasi‐experimental design, employing pre‐test and posttest group comparisons to examine the effectiveness of the NMT intervention in diabetic patients [16]. Participants were individuals diagnosed with diabetes, who underwent an initial assessment using the diabetic neuropathy syndrome (DNS) instrument. A DNS score above one indicated the presence of diabetic neuropathy. Subsequently, participants engaged in a structured NMT program comprising six sessions held every 4 days. During each session, participants' ankle‐brachial index (ABI), Toronto Clinical Neuropathy Scoring System (TCNSS), and Diabetic Neuropathy Examination (DNE) were assessed to monitor their progress and response to the intervention. Additionally, platelet indices, including Platelet Distribution Width (PDW), Mean Platelet Volume (MPV), Plateletcrit (PCT), and Mean Platelet Volume (MPV), were measured both before (pre‐) and after (post‐) the exercise program to investigate any potential changes in thrombocyte indices. The pre‐test and posttest results from these assessments served as the primary data for the research analysis. A visual representation of the study procedure, outlining the various assessments and intervention sessions, is depicted in Figure 1. This study received ethical approval from the Health Research Ethics Committee of the Faculty of Medicine, University of Muhammadiyah Surakarta (Approval No. 180/8.02.01/KEPK/XI/2022). All participants provided written informed consent before inclusion in the study, in accordance with the Declaration of Helsinki.

### 2.1 Participants

Participants were recruited from the PROLANIS program, which is implemented at four community health centers in Pekalongan, Indonesia. Eligible participants included adults diagnosed with diabetic neuropathy (DN) who were willing and able to provide written informed consent. Exclusion criteria included individuals with diabetes mellitus who had concurrent conditions known to exacerbate neuropathy risk, such as acute thrombosis, active cancer or metastasis, phlebitis, acute congestive heart failure, ongoing infections, recent muscle trauma, ulceration, or edema related to heart failure. Patients with any additional diabetes complications outside the standard criteria of the PROLANIS program were also excluded. All inclusion and exclusion criteria adhered to national standards established by the Indonesian Ministry of Health [17]. In addition, the power analysis of this study was conducted using G*Power version 3.1.9.7. Setting an effect size of 0.8, a significance level (alpha) of 0.1, and a desired power of 0.95, the analysis determined that a minimum sample size of 19 was necessary. It is noteworthy that the study ultimately utilized 23 samples, exceeding the calculated requirement. We have recruited a sample size of 132 participants for this study. Out of the 132 individuals with diabetes who expressed interest, 92 were identified with a DNS score exceeding 1 as per WHO criteria [18]. Subsequently, 23 individuals agreed to partake in the study, ensuring a rigorous selection process of participants meeting diabetic neuropathy criteria and displaying readiness for research involvement. Focusing on PROLANIS program participants facilitated a diverse representation of individuals with diabetes from the community, thereby enhancing the study's findings' generalizability to the broader population [19].

### 2.2 The NMT Procedure

The NMT method involves several steps for decompression application. Firstly, the tape is cut into one fan‐shaped pieces, each measuring 20–30 cm in length and 1 cm in width. Before application, the patient's foot area is thoroughly cleaned using water swabs to ensure proper adhesion. Care is taken to avoid touching the adhesive side of the tape to maintain its effectiveness. With the patient positioned prone and the knee flexed at a 90‐degree angle, and the ankle in a dorsiflexed position, the tape is carefully applied to the sole of the foot, starting from the heel and extending towards the toes. Strips of tape are then cut and applied individually to each metatarsal bone, ensuring proper coverage. Evaluation of the tape's placement is conducted by observing any wrinkles on the sole of the foot, indicating appropriate decompression and adhesion. Subsequently, the patient is repositioned in a supine position, with the foot in an inverted position for further taping. The base tape is applied above the lateral malleolus, followed by crisscrossing application of tape to each toe, and then above the medial malleolus with the foot in an everted position. Wrinkles in the tape on the back of the foot are observed to ensure correct decompression application [20].

### 2.3 Assessment of DNS, ABI, TCNSS, and DNE

Data collection for the evaluation of DNS involved administering the DNS instrument to each participant. This tool comprises a series of questions and clinical examinations aimed at assessing neuropathy symptoms' presence and severity in diabetic individuals. Participants responded to specific inquiries regarding symptoms like tingling sensations, numbness, and pain in the extremities. Additionally, clinical examinations, including sensory and reflex tests, were conducted to objectively evaluate neuropathy symptoms [21].

The TCNSS was employed to evaluate neuropathy severity and progression among participants. This standardized scoring system integrates various clinical parameters, including neurological examinations, reflexes, and sensory perception tests, to quantify neuropathic impairment's extent. Each participant underwent a comprehensive TCNSS assessment, with scores recorded to monitor changes in neuropathy severity over time [22].

ABI measurements were obtained to assess peripheral arterial circulation and detect potential PAD. ABI, a noninvasive vascular test, compares blood pressure measurements in the ankles and arms. A lower ABI value indicates impaired blood flow to the lower extremities, indicative of PAD. Participants underwent ABI measurements using Doppler ultrasound, and readings were documented for further analysis [23].

The DNE was conducted to assess various neuropathy aspects, including sensory perception, reflexes, and muscle strength. This comprehensive examination comprised a series of clinical tests, such as monofilament testing for sensation assessment, ankle reflex testing for nerve function evaluation, and muscle strength assessments. Each participant underwent a thorough DNE examination, and findings were recorded to assess neuropathy status and intervention response [24]. Trained healthcare professionals conducted all assessments following standardized protocols to minimize variability and bias. Data collected from the DNS, TCNSS, ABI, and DNE assessments were systematically recorded and stored for subsequent analysis.

### 2.4 Assessment of Hematological Parameters

Platelet indices, including PDW, MPV, PCT, and PLT, were assessed using an Automated Hematology Analyzer. This device employs advanced technology to analyze blood samples and quantify various hematological parameters, including platelet characteristics. As blood samples pass through the analyzer's aperture, changes in impedance are detected, allowing for the precise determination of platelet count and indices. Participants' blood samples were collected via standard venipuncture methods and anticoagulated with ethylene diamine tetra acetic acid. The samples were then processed and analyzed by the Automated Hematology Analyzer to derive platelet indices. The analyzer automatically measures platelet parameters based on the size, volume, and distribution of platelets in the sample. The recorded platelet indices for each participant at both pre‐test and posttest stages were used to evaluate changes in thrombocyte characteristics throughout the intervention.

### 2.5 Analyzing Molecular Mechanisms of DN Development

Several studies have discussed the relationship between thrombocytes and DN, highlighting the importance of platelet indices in this context [25, 26, 27, 28, 29, 30]. However, to the best of our knowledge, there has not been a genomic study directly linking DN with thrombocyte behavior. To elucidate the molecular mechanisms associated with the development of DN, we extracted data from the GEO databases. Specifically, we utilized the GSE185011 data set as the basis for our analysis [31]. Differential Expression Genes (DEGs) were extracted from GSE185011 using GEO2R as the analysis tool. We employed Type 2 Diabetes Mellitus (T2DM) as the control group for DN development. GSE185011 is a data set containing gene expression profiles from patients with T2DM and DN, allowing for the identification of DEGs implicated in DN pathogenesis. The use of T2DM as a control group enables the identification of genes specifically associated with DN development, thereby providing insights into the molecular mechanisms underlying this condition.

### 2.6 Data Analysis

Statistical analysis in this study was conducted using R software (version 2023.03.0; R Core Team), incorporating relevant packages (stats and psych). Data visualization was performed using the ggplot2 and corrplot package, while OriginPro software was also employed for enhanced graphical representation. Power analysis to determine sample adequacy was conducted using G*Power (version 3.1.9.7). Pre‐specified analyses included paired and independent sample *t*‐tests, as well as Analysis of Covariance (ANCOVA) and linear regression modeling to adjust for confounders. The Cochrane–Bantel test was used for dichotomous comparisons. All statistical tests were two‐sided, with a priori significance level set at *α* = 0.05.

## 3 Result

### 3.1 Assessment of Reliability Using Cronbach's Alpha Coefficient

We describe the validation procedure for the measurement instruments employed in the study. We assessed the reliability of key assessment tools, namely the ABI, TCNSS, and DNE, using Cronbach's Alpha coefficient. This coefficient is a widely recognized measure for assessing the internal consistency of measurement scales. Our analysis yielded Raw Alpha coefficients of 0.92 for ABI, 0.87 for TCNSS, and 0.93 for DNE, indicating robust internal consistency among the items within each instrument. These findings highlight the reliability of our selected assessment tools in accurately evaluating peripheral arterial circulation and neuropathy severity among the participants of the study. Further details regarding the validation process are provided in Supporting Information Table.

### 3.2 Characteristics of Study Participants

Table 1 presents the characteristics of the respondents, demonstrating a diverse representation of individuals with diabetes involved in the study. The cohort consisted of 23 participants, with a gender distribution of three males and 20 females. Age classification revealed that nine individuals belonged to the adult group, while 14 were categorized as geriatric. Regarding the duration of illness, nine participants had been diagnosed for less than 5 years, while 14 had been ill for more than 5 years. Additionally, based on obesity status, nine individuals were classified as having overweight, whereas 14 participants were considered obese.

**Table 1:** Characteristics of the respondent.

			Average score					
		*n*	ABI (Mean ± SD)	*p* value	TCNSS (Mean ± SD)	*p*‐value	DNE (Mean ± SD)	*p* value
Gender								
	Male	3	0.976 ± 0.120	0.214	6.000 ± 0.577	0.205	5.000 ± 0.000	0.485
	Female	20	1.229 ± 0.210		6.936 ± 1.238		5.876 ± 0.619	
Age								
	Adult	9	0.712 ± 0.172	0.340	6.777 ± 1.202	0.145	5.111 ± 0.601	0.469
	Geriatric	14	0.914 ± 0.215		7.214 ± 1.114		5.428 ± 0.513	
Duration of illness								
	> 5 years	4	0.895 ± 0.231	0.301	6.947 ± 0.866	0.400	5.263 ± 0.500	0.307
	< 5 years	19	0.922 ± 0.208		7.500 ± 1.140		5.500 ± 0.577	
Obesity								
	Overweight	20	0.825 ± 0.190	0.325	7.200 ± 1.223	0.400	5.000 ± 0.520	0.307
	Obese	3	0.943 ± 0.110		6.700 ± 0.577		5.350 ± 0.265	

Adults were characterized as individuals aged 18 years and older, whereas geriatric participants encompassed those aged 60 years and above. Additionally, participants' body mass index (BMI) was employed to categorize them into either overweight or obese groups. Overweight classification was attributed to individuals with a BMI below 25, signifying a comparatively lower body weight, while those with a BMI exceeding 25 were designated as obese [32].

**Figure 2:**
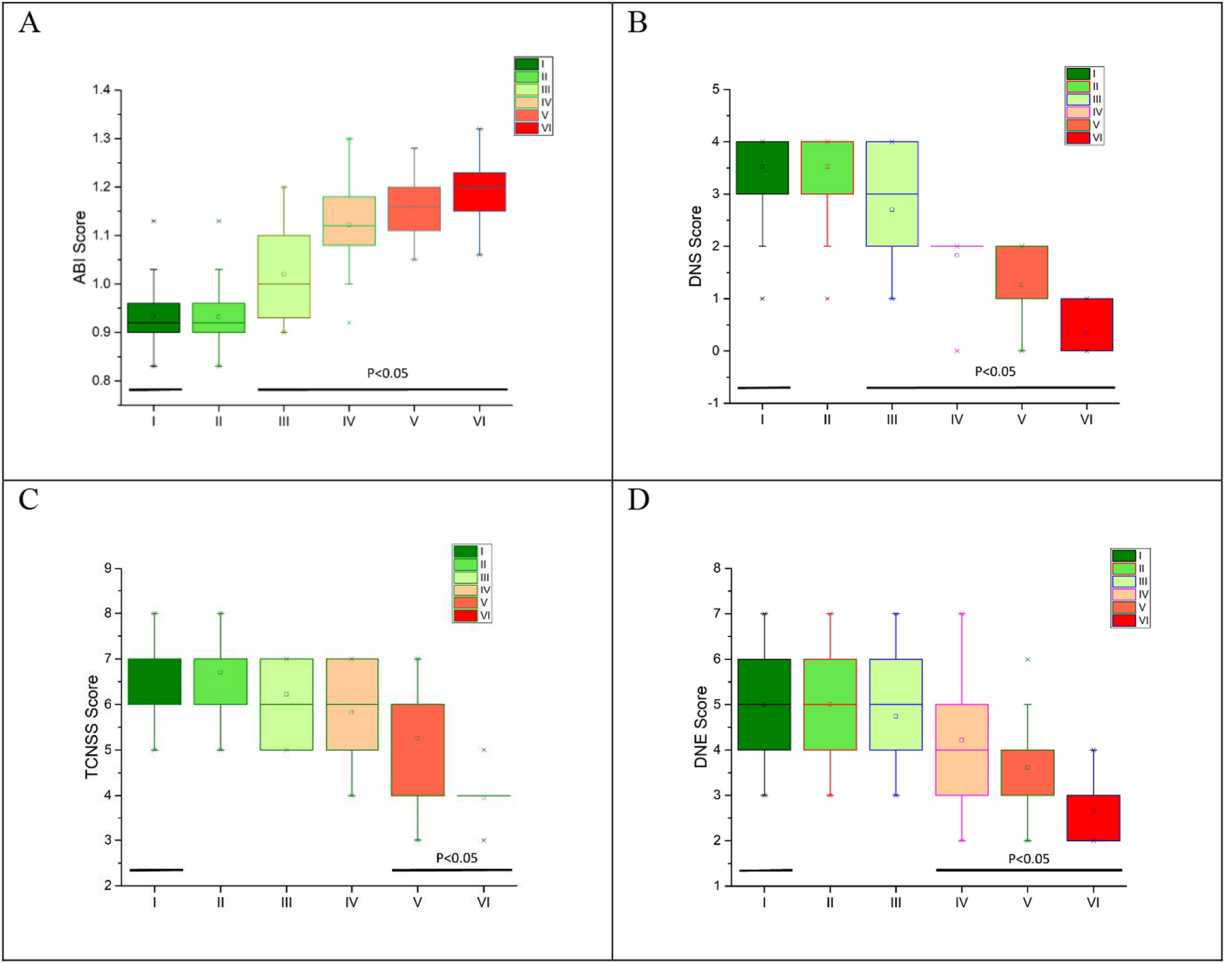
The Impact of NMT Intervention on ABI, DNS, TCNSS, and DNE Values Throughout Treatment Sessions (A) ABI, (B) DNS, (C) TCNSS, (D) DNE.

Subsequently, we proceeded with analyses to assess the impact of the NMT intervention, conducted every 4 days over six sessions, on the values of ABI, DNS, TCNSS, and DNE. As illustrated in Figure 2A–D panel, the intervention had a statistically significant effect on all three parameters. Additionally, we aimed to pinpoint the specific intervention session(s) at which the NMT produced significant changes in outcomes. This determination is crucial for determining the minimum number of treatments necessary for the intervention to achieve meaningful results. Those Figure further indicates that significant alterations in ABI and DNS values occurred start from the third intervention session, while TCNSS and DNE values exhibited significant changes start from the fifth and fourth respectively intervention session. These findings offer valuable insights into the temporal dynamics of the intervention's efficacy and provide guidance for optimizing its duration and frequency in the management of peripheral artery disease and diabetic neuropathy.

### 3.3 Exploring Relationships Among ABI, TCNSS, and DNE

**Figure 3:**
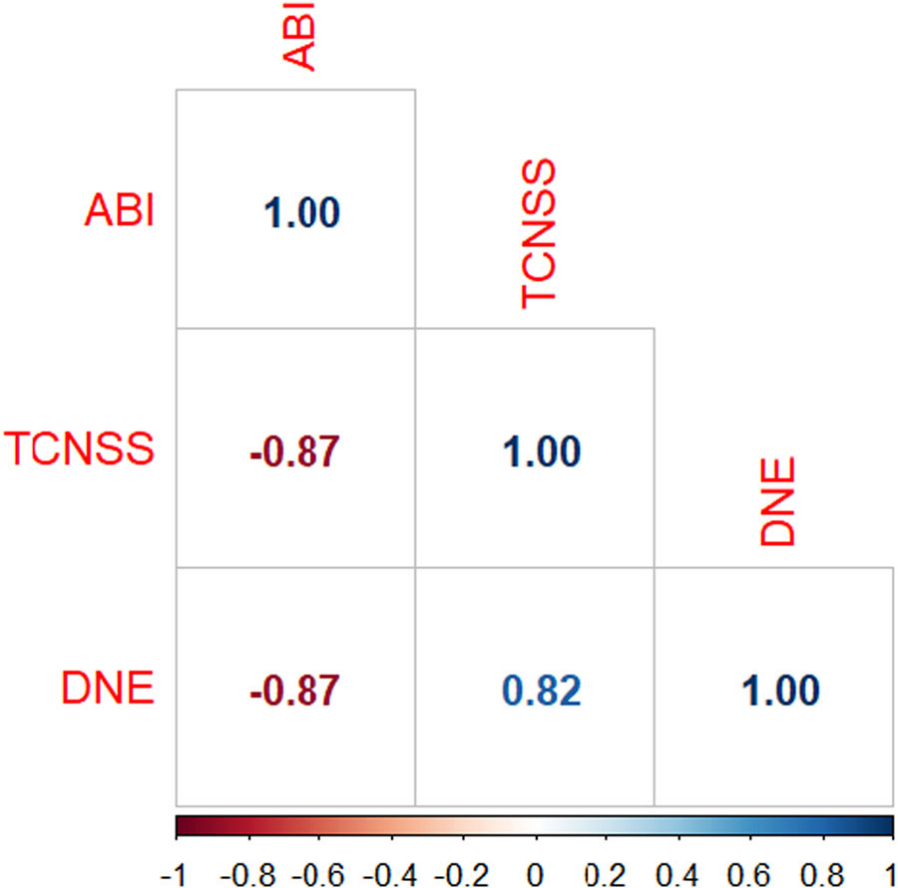
Correlation analysis of ABI, TCNSS, and DNE Scores. Spearman correlation coefficients range from −1 to +1, where +1 indicates a perfect positive linear relationship, −1 indicates a perfect negative linear relationship, and 0 indicates no linear relationship.

The Spearman correlation coefficient serves as a statistical metric for assessing the strength and direction of the linear relationship between two continuous variables [33]. In this study, we employed Spearman correlation to explore the associations among ABI, TCNSS, and DNE, as depicted in Figure 3. Our analysis unveiled distinctive correlation patterns among these variables. Notably, ABI displayed a negative correlation with both TCNSS and DNE scores, indicating that higher ABI values were linked to lower TCNSS and DNE scores. Conversely, TCNSS exhibited a positive correlation with DNE scores, suggesting that higher TCNSS values corresponded to higher DNE scores. These findings offer valuable insights into the interplay among peripheral arterial circulation, neuropathy severity, and diabetic neuropathy examination outcomes, highlighting the effectiveness of Spearman correlation in unraveling intricate relationships within the study variables.

### 3.4 Effects of NMT Intervention on Platelet Indices

The impact of the NMT intervention on alterations in platelet indices (PDW, MPV, PLT, and PCT) before and after intervention was explored. Employing the generalized linear model analysis technique, we assessed these effects while considering potential confounders like age, obesity, and duration of illness. The findings from this analysis are outlined in Table 2, demonstrating noteworthy effects of the NMT treatment on PDW, MPV, and PLT indices.

**Table 2:** Effects of NMT on Platelet Indices (PDW, MPV, PLT, and PCT) Pre and Post‐Intervention, Controlling for Age, Obesity, and Duration of Illness.

	*p* value			
	PDW	MPV	PLT	PCT
Pre‐	0.157	0.0560	0.00296*	0.0194*
Obesity	0.323	0.0555	0.96304	0.2901
Age	0.991	0.5491	0.79383	0.7393
Duration of illness	0.710	0.9098	0.04724*	0.1552

* *p* < 0.05.

### 3.5 Analysis of DEGs Reveals Molecular Insights Into DN Pathogenesis

To elucidate the molecular mechanisms underlying these observed clinical improvements, we conducted a genomic analysis using data from the GEO database, specifically the data set GSE185011. In the data set GSE185011, comprising gene expression profiles from patients with T2DM and DN, a total of 10 respondents were included, with five individuals representing each group. Differential Expression Genes (DEGs) analysis was conducted using GEO2R [34]. The results of this analysis, illustrated in Table 3, delineate the DEGs identified in the comparison between T2DM and DN groups.

**Table 3:** Genes associated with DNE development.

GeneID	Symbol	Function	log2
7779	SLC30A1	Solute carrier family 30 member 1	0.677
28622	TRBJ2‐7	T cell receptor beta joining 2–7	−1.074
10439	OLFM1	Olfactomedin 1	2.733
6934	TCF7L2	Transcription factor 7 like 2	0.932
23263	MCF2L	MCF.2 cell line derived transforming sequence like	−1.035
100653515	CEP295NL	CEP295 N‐terminal like	0.874
388550	CEACAM22P	CEA cell adhesion molecule 22, pseudogene	−3.036
128553	TSHZ2	Teashirt zinc finger homeobox 2	−1.104
57595	PDZD4	PDZ domain containing 4	0.759

**Figure 4:**
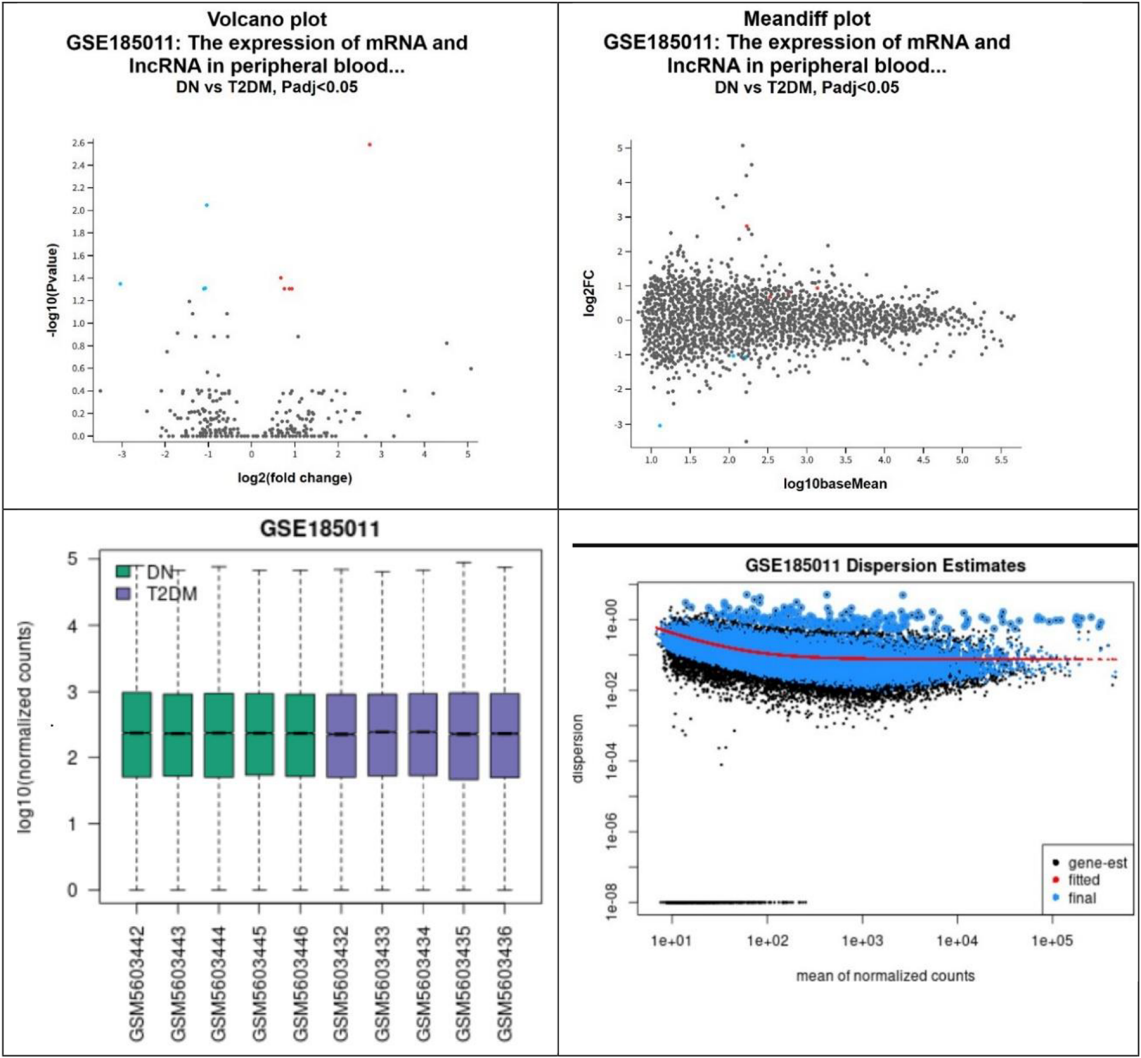
The detailed DEGs result analysis. Left Upper Panel: The volcano plot displays the differential expression of genes between diabetic neuropathy (DN) patients and the control group (T2DM patients without DN). The x‐axis represents the log2 fold change, and the y‐axis represents the ‐log10 *p*‐value. Red dots indicate significantly upregulated genes, while blue dots indicate significantly downregulated genes (*p* < 0.05, fold change > 2). Right Upper Panel: The MA (Mandiff) plot visualizes the log‐intensity ratios (M) versus the mean log‐intensity (A) of genes between DN patients and the control group. Genes with significant differential expression are highlighted, with red indicating upregulation and blue indicating downregulation. Bottom Left Panel: The within‐array normalization results ensure that the data are properly adjusted for technical variations across arrays. This normalization process is crucial for the accurate identification of Differential Expression Genes (DEGs). Bottom Right Panel: The dispersion estimate plot shows the dispersion of gene expression levels, reflecting the variability in gene expression across samples. Proper dispersion estimates are essential for reliable differential expression analysis.

Notably, the DEGs encompass genes implicated in various biological processes and pathways relevant to neuropathy pathogenesis. This assessment allows for the elucidation of key molecular players involved in DN progression and provides valuable insights into potential therapeutic targets for managing this condition. The detailed DEGs result analysis was depicted in Figure 4.

## 4 Discussions

In this study, we examined the effectiveness of the NMT intervention in addressing both PAD and DN in individuals with diabetes. Upon examining gender distribution, the average ABI, TCNSS, and DNE scores in male participants were 0.976, 6.000, and 5.000, respectively, compared to 1.229, 6.936, and 5.876 in female participants. Statistical comparisons using the independent *t*‐test yielded P values of 0.21 for ABI, 0.21 for TCNSS, and 0.49 for DNE, indicating no statistically significant differences between genders. Similarly, when comparing age groups, adults showed mean ABI, TCNSS, and DNE scores of 0.712, 6.777, and 5.111, while geriatric individuals had scores of 0.914, 7.214, and 5.428. The corresponding P values were 0.34, 0.15, and 0.47, respectively. In relation to disease duration, participants with more than 5 years of illness had ABI, TCNSS, and DNE averages of 0.895, 6.947, and 5.263, respectively, versus 0.922, 7.500, and 5.500 for those with less than 5 years, with *p* values of 0.30, 0.40, and 0.31, respectively. These findings suggest no statistically significant differences in these parameters across the analyzed subgroups. These outcomes are consistent with the findings reported by Stuart et al. in their 2007 study [35] and other studies as well [36]. Further, a recent study highlighted the significant association between ABI values and the risks of type 2 diabetes complications in individuals who were asymptomatic for PAD. The lower ABI values were correlated with an increased risk of diabetic complications, including neuropathy, even in the absence of PAD symptoms [36].

It is important to note that there is limited literature investigating the impact of NMT on ABI scores, particularly in the context of diabetic neuropathy [37]. While several studies have suggested that NMT stimulate and restrict motion neuromuscular, especially in other populations, such as athletes or individuals with musculoskeletal conditions, there is scarce evidence regarding its effects on ABI scores in diabetic neuropathy patients. Specifically concerning hematological aspects, to the best of our knowledge, no studies have reported on this association. The existing literature on NMT primarily focuses on its role in enhancing neuromuscular function, reducing pain, and improving range of motion. Studies often highlight its application in conditions such as sports injuries, musculoskeletal disorders, and rehabilitation settings [38, 39, 40, 41, 42]. However, its specific effects on vascular parameters, such as ABI scores, remain relatively understudied, especially in diabetic neuropathy patients. Given the potential benefits of NMT in improving blood circulation and reducing pain, further research is warranted to explore its efficacy in diabetic neuropathy patients. This study contributes to this area by demonstrating a significant reduction in both TCNSS and DNE scores following the NMT intervention. This observed effect can be attributed to the intervention's ability to augment blood flow to the peripheral regions, leading to improvements in TCNSS and DNE scores. Essentially, the enhancements in ABI reflect better blood circulation to the lower extremities, signifying improved vascular health, while the decreases in TCNSS and DNE scores suggest alleviation of neuropathy‐related symptoms. These findings are corroborated by the data presented in Figure 2, validating our results.

Furthermore, the findings depicted in Figure 2 elucidate the timing at which NMT intervention yielded significant results in DNS, ABI, TCNSS, and DNE scores. This temporal aspect is crucial, especially concerning its implications as a guide for healthcare providers in determining the frequency and duration of NMT intervention. It is noteworthy that the identification of the optimal timing for administering NMT is essential due to the discomfort that some individuals may experience during its application. The data presented in Figure 2 provide valuable insights into the progression of changes in DNS, ABI, TCNSS, and DNE scores over the course of the NMT intervention. By identifying the intervention sessions at which significant improvements are observed in these scores, healthcare providers can better tailor the treatment regimen to maximize its effectiveness while minimizing discomfort and inconvenience for patients. For instance, knowing that significant improvements in TCNSS scores occur at the third intervention session, and in DNE scores at the fourth session, enables healthcare providers to plan NMT sessions accordingly, optimizing the timing for achieving desired outcomes. Moreover, understanding the temporal dynamics of NMT efficacy can aid healthcare providers in setting realistic expectations for patients undergoing the intervention. By informing patients about the anticipated timeline for observing improvements in neuropathy symptoms and vascular parameters, healthcare providers can enhance patient satisfaction and adherence to the treatment plan. Additionally, this knowledge enables healthcare providers to monitor patients' progress effectively throughout the intervention period and make timely adjustments to the treatment regimen as needed.

The correlation analysis revealed intriguing connections among ABI, TCNSS, and DNE scores. Specifically, ABI demonstrated a negative correlation with TCNSS and DNE scores, whereas TCNSS exhibited a positive correlation with DNE scores. These associations underscore the interconnected nature of peripheral arterial circulation, neuropathy severity, and diabetic neuropathy examination outcomes. Such insights contribute to our comprehension of the intricate pathophysiological mechanisms involved in PAD and DN among diabetic patients. Studies conducted by Takashi et al. have provided valuable insights into the link between platelet aggregability and PAD [43]. Their findings validate that patients with PAD exhibit increased platelet aggregability, and the extent of platelet aggregation is closely linked to ABI values. This relationship emphasizes the clinical importance of platelet function in the pathophysiology of PAD and highlights the potential use of ABI as a surrogate marker for evaluating platelet activity in these patients. Understanding the interplay between platelet function and vascular health is pivotal for devising effective therapeutic approaches for PAD management, with implications for mitigating thrombotic events and enhancing patient outcomes.

The results presented in Table 2 show the effects of Neuromuscular Taping (NMT) on platelet indices, including PDW, MPV, PLT, and PCT, before and after the intervention, while accounting for age, obesity, and duration of illness. Statistically significant improvements were observed in PLT and PCT following the intervention, indicating a measurable impact of NMT on these parameters. In contrast, changes in PDW and MPV were not statistically significant. Further analysis revealed that age and obesity did not significantly influence any of the platelet indices. However, duration of illness was found to have a significant effect on PLT, suggesting a possible association between longer disease duration and changes in platelet count. These findings suggest that NMT may exert a modulatory effect on specific platelet indices, particularly PLT and PCT, among individuals with diabetes. Overall, these findings contribute to our understanding of the potential hematological effects of NMT in diabetic patients, highlighting its potential as a therapeutic intervention for managing platelet‐related complications in diabetes.

In addition to investigating the clinical effects of interventions such as NMT, we are also concerned with elucidating the molecular mechanisms underlying the development of DN. Through a series of analyses, we identified several genes implicated in DN development. These genes include SLC30A1, TRBJ2‐7, OLFM1, TCF7L2, MCF2L, CEP295NL, CEACAM22P, TSHZ2, and PDZD4. Each of these genes plays a distinct role in biological pathways that have been associated with the pathogenesis and progression of DN. For instance, SLC30A1 is involved in ion transport processes and has been linked to neuronal function and oxidative stress modulation, both of which are critical factors in DN development [44]. Similarly, OLFM1 is implicated in neuronal development and may contribute to the regulation of synaptic plasticity, which is perturbed in diabetic neuropathy [45]. Additionally, genes such as TCF7L2 have been associated with glucose homeostasis and insulin signaling pathways [46], suggesting a potential role in the metabolic dysregulation observed in diabetes and its complications. Furthermore, the identification of CEP295NL, CEACAM22P, and TSHZ2 underscores the importance of exploring novel genetic factors that may contribute to DN pathogenesis [47, 48, 49]. While their specific roles in DN development require further investigation, their differential expression patterns in diabetic neuropathy suggest potential involvement in disease mechanisms. By elucidating the molecular pathways underlying DN development, we can develop more targeted and effective strategies for prevention and treatment, ultimately improving outcomes for individuals affected by this debilitating condition.

There are several limitations that should be acknowledged in this study. Firstly, the sample size was relatively small, which may restrict the applicability of the findings to broader populations of diabetic neuropathy patients. Increasing the sample size would enhance the statistical power and reliability of the results. Additionally, the follow‐up duration was short, limiting our ability to evaluate the long‐term effects of the NMT intervention on peripheral artery disease and diabetic neuropathy outcomes. Moreover, the study design was quasi‐experimental, which prevents making causal inferences and may introduce biases inherent to non‐randomized studies. Future research using randomized controlled trials with extended follow‐up periods and larger sample sizes is necessary to confirm our findings and understand the underlying mechanisms of the NMT intervention in managing peripheral artery disease and diabetic neuropathy. Although we controlled for certain confounding factors like age, obesity, and duration of illness in the analyses, there could be other unmeasured confounders that might influence the results. Lastly, the study focused on diabetic neuropathy patients in a specific geographical region, which could limit the generalizability of the findings to other populations or settings. Future studies involving more diverse patient populations and settings would offer a more comprehensive understanding of the effectiveness and applicability of the NMT intervention in managing peripheral artery disease and diabetic neuropathy.

### 4.1 Implications on Clinical Practice

The implications of studies conducted with physiotherapy, particularly using the NMT decompression method, hold significant promise for individuals with diabetes, a condition notorious for its multifaceted impact on various bodily systems. In diabetic patients, inflammation is a common occurrence, contributing to a cascade of complications that adversely affect their quality of life [50]. Among these complications, one notable effect is the decrease in skin elasticity, which not only compromises the skin's protective barrier but also impairs sensory function, particularly in the lower extremities [51]. This diminished sensory perception in the feet can lead to heightened vulnerability to injuries and infections, potentially culminating in severe complications such as diabetic neuropathy and foot ulcers. Moreover, inflammation‐induced swelling in diabetic individuals often results in limited mobility, as the joints become stiff and the range of motion becomes restricted. This limitation, compounded by muscle weakness caused by inflammation, poses significant challenges to daily activities and compromises overall physical function [52]. Additionally, diabetic neuropathy frequently manifests as tingling sensations or numbness in the feet, further exacerbating mobility issues and increasing the risk of falls or accidents [53]. Furthermore, the diminished weight‐bearing capacity due to inflammation‐related discomfort can disrupt the natural gait pattern, leading to altered biomechanics and increased strain on other joints and muscles [54].

Previous studies have elucidated the intricate relationship between skin elasticity and thrombocytes. These investigations have highlighted the pivotal role of thrombocytes, also known as platelets, in maintaining the integrity and resilience of the skin [55, 56, 57]. Thrombocytes not only play a crucial role in hemostasis and wound healing but also actively participate in the synthesis and remodeling of collagen, a fundamental structural protein in the skin's extracellular matrix [58]. Consequently, disruptions in thrombocyte function, such as thrombocytopenia or aberrant platelet aggregation, can compromise collagen metabolism and lead to diminished skin elasticity. This association underscores the importance of optimizing thrombocyte activity for preserving skin health and elasticity, offering potential avenues for therapeutic intervention in conditions affecting skin integrity. Moreover, our study specifically found that NMT significantly increases the number of PLT and PCT. This finding not only supports the beneficial effects of NMT on thrombocyte function but also suggests a potential mechanism by which NMT may enhance skin elasticity. By promoting an increase in platelet count and activity, NMT may facilitate enhanced collagen synthesis and remodeling, thereby contributing to improved skin elasticity and overall skin health. These insights underscore the potential of NMT as a therapeutic modality for addressing both thrombocyte‐related issues and skin elasticity concerns, offering a holistic approach to promoting skin health and resilience. In summary, the integration of physiotherapy interventions, especially those leveraging innovative techniques like NMT decompression, holds immense potential in ameliorating the deleterious effects of inflammation in diabetic patients. By addressing the interconnected components of inflammation‐related complications comprehensively, physiotherapy not only alleviates symptoms but also fosters resilience, functional independence, and improved quality of life for individuals living with diabetes.

This study has several limitations that should be acknowledged. First, the relatively small sample size may limit the generalizability of the findings to broader populations. Larger multicenter trials are needed to validate the observed effects. Second, most study participants were female, which may have introduced a gender bias. It is important to consider that pharmacological responses, including those to oral antidiabetic drugs such as thiazolidinediones and sulfonylureas, have been shown to differ between males and females across various ethnic groups [59]. Therefore, future studies should aim for a more balanced gender distribution to better understand potential sex‐specific responses to both neuromuscular interventions and concurrent medical therapies.

## 5 Conclusion

In conclusion, this study provides valuable insights into the efficacy of the NMT intervention in managing PAD and DN among individuals with diabetes. Our findings demonstrate significant improvements in ABI, TCNSS, and DNE scores following the NMT intervention, highlighting its potential in enhancing peripheral arterial circulation and ameliorating neuropathy symptoms. In addition, further research is needed to explore the roles of specific biomolecules implicated in diabetic neuropathy development, such as SLC30A1, TRBJ2‐7, OLFM1, TCF7L2, MCF2L, CEP295NL, CEACAM22P, TSHZ2, and PDZD4. However, several limitations should be acknowledged, including the relatively small sample size, short follow‐up duration, and quasi‐experimental study design. Future research employing randomized controlled trials with larger sample sizes and longer follow‐up periods is warranted to validate our findings and elucidate the underlying mechanisms of the NMT intervention.

## Author Contributions

**Nurul Aktifah:** conceptualization, writing – original draft, methodology. **Firman Faradisi:** investigation, software, formal analysis, data curation. **Muhammad Ghilang Maulud Setyawan:** investigation, validation, visualization, data curation. **Nuniek Nizmah Fajriyah:** writing – review and editing, funding acquisition, project administration. **Eko Mugiyanto:** conceptualization, methodology, project administration, writing – review and editing. **Umi Budi Rahayu:** resources, formal analysis, writing – review and editing, supervision. All authors have read and approved the final version of the manuscript. All authors have read and approved the final version of the manuscript. Eko Mugiyanto (Corresponding Author) had full access to all the data in this study and takes full responsibility for the integrity of the data and the accuracy of the data analysis.

## Conflicts of Interest

The authors declare no conflicts of interest.

## Transparency Statement

Eko Mugiyanto affirms that this manuscript is an honest, accurate, and transparent account of the study being reported; that no important aspects of the study have been omitted; and that any discrepancies from the study as planned have been explained.

## Supporting information

Supplementary paper 2 rev1.

## Data Availability

The data that support the findings of this study are available from the corresponding author upon reasonable request. The data that support the findings of this study are available from the corresponding author, Eko Mugiyanto, upon reasonable request. Due to privacy and ethical restrictions, some data may not be publicly available.
