# Supplementary material for: Assessing the Impact of Neuromuscular Taping on Thrombocyte Indices in Diabetic Neuropathy Patients With Peripheral Artery Disease: A Cross‐Sectional Study
Source: Health Sci Rep. 2025 Jul 20;8(7):e70919. doi: 10.1002/hsr2.70919 (PMC12277234; doi:10.1002/hsr2.70919)
Supplement: Supplementary file 1 — Supplementary paper 2 rev1. [file HSR2-8-e70919-s001.docx]

**Supplementary**

**Assessing the Impact of Neuromuscular Taping on Thrombocyte Indices in Diabetic Neuropathy Patients with Peripheral Artery Disease**

Nurul Aktifah^1^, Firman Faradisi^2^, Muhammad Ghilang Maulud Setyawan^1^,

Nuniek Nizmah Fajriyah^2^, Eko Mugiyanto^3,4*^, Umi Budi Rahayu^5^

1. Undergraduate Program in Physiotherapy, University of Muhammadiyah Pekajangan Pekalongan, Indonesia
2. Vocational Program in Nursing, University of Muhammadiyah Pekajangan Pekalongan, Indonesia
3. Department of Pharmacy, University of Muhammadiyah Pekajangan Pekalongan, Indonesia
4. Reka Institute of Science and Technology, Indonesia
5. Faculty of Health Sciences, Universitas Muhammadiyah Surakarta, Surakarta, Indonesia

Corresponding author: Eko Mugiyanto

giyan77@gmail.com

**Supplementary**

| **Supplementary Table.** Validation Results of Measurement Instruments Using Cronbach's Alpha Coefficient |
| --- |
| \| Parameter \| ABI \| TCNSS \| DNE \| \| --- \| --- \| --- \| --- \| \| Raw Alpha \| 0.92 \| 0.87 \| 0.93 \| \| Std.Alpha \| 0.92 \| 0.87 \| 0.93 \| \| G6(smc) \| 0.93 \| 0.93 \| 0.96 \| \| Average R \| 0.36 \| 0.24 \| 0.40 \| \| S/N \| 12.00 \| 6.70 \| 14.00 \| \| ASE \| 0.03 \| 0.05 \| 0.02 \| \| Mean \| 1.10 \| 5.60 \| 4.30 \| \| SD \| 0.08 \| 0.68 \| 0.86 \| \| Median R \| 0.38 \| 0.24 \| 0.38 \| |
